# The effect of *BRAF^V600E^* mutation on radioiodine therapy in patients with papillary thyroid carcinoma: a meta-analysis and systematic review

**DOI:** 10.3389/fendo.2025.1665545

**Published:** 2025-09-23

**Authors:** Bin Wang, Xiao-Xia Cen, Bo-Rui Zhang, Wei Zhang

**Affiliations:** Department of Thyroid, Breast and Hernia Surgery, Changzheng Hospital Affiliated to Navy Medical University, Huangpu, Shanghai, China

**Keywords:** papillary thyroid carcinoma, radioactive iodine refractoriness, BRAFV600E mutation, therapeutic response, meta-analysis

## Abstract

**Objective:**

The *BRAF^V600E^* mutation is one of the most common genetic alterations in papillary thyroid cancer (PTC) and is widely recognized as a factor of poor prognosis. Radioactive iodine (RAI) therapy is recommended after thyroidectomy for patients with high-risk level PTC or distant metastatic PTC. However, the association between *BRAF^V600E^* mutation and RAI refractoriness remains controversial and requires additional investigation. This meta-analysis was conducted to evaluate the impact of *BRAF^V600E^* mutation on the curative effect of RAI therapy.

**Materials and methods:**

Electronic searches for relevant studies were performed in the databases of PubMed, EMBASE, and the Science Citation Index databases, with publication date after January 2005. The ^131^I uptakes status, response to RAI therapy and recurrence of RAI therapy were extracted and compared using meta-analysis.

**Results:**

We included 14 eligible studies incorporating 2890 PTC patients, of which 1966 were patients with *BRAF^V600E^* mutation. The pooled analysis indicated that *BRAF^V600E^* mutation was strongly associated with the loss of iodine avidity (P = 0.0003) in PTC patients, especially in recurrent individuals (P<0.0001). However, *BRAF^V600E^* mutation had no significant association on the clinical response or recurrence rate of RAI therapy (P = 0.27 and P = 0.29, respectively).

**Conclusion:**

This meta-analysis confirmed the role of *BRAF^V600E^* mutation in deterioration of the ability of RAI uptake but provided insufficient evidence to demonstrate that *BRAF^V600E^* mutation might impact the clinical response and recurrence after postsurgical RAI therapy for PTC patients.

## 1 Introduction

Papillary thyroid carcinoma (PTC) is the most common subtype of thyroid malignancy (1). PTC is an indolent disease that usually has a favorable prognosis with 10-year overall survival rates higher than 90% after appropriate treatment (2). According to the guidelines in 2015, surgical resection is the preferred treatment and widely used in clinical practice for PTC (3). Radioactive iodine (RAI) therapy is not routinely recommended after thyroidectomy for patients with PTC (3). However, a small subset of PTC patients with high-risk features (such as extrathyroidal extension, macroscopic lymph node metastases, aggressive histology as well), or even clinically significant distant metastases are classified as intermediate-risk or high-risk PTC patients. For these individuals, RAI using radioiodine (^131^I) may be beneficial for eliminating residual thyroid tissue as well as persistent thyroid cancer cells, thereby reducing disease recurrence. Approximately 15–20% of patients experience recurrence. One-third of these recurrent patients demonstrate loss of radioiodine avidity, leading to radioactive iodine refractoriness (RAIR), thus rendering the most critical tool available for PTC treatment ineffective (4). Patients with excellent responses to RAI exhibit a favorable prognosis with a low recurrence rate. Conversely, those resistant to RAI have higher risks of locoregional recurrences and metastases during the follow-up period, resulting in an elevated mortality rate. It is still a clinical challenge for clinicians to evaluate the ability of ^131^I uptake at an early stage to tailor individual therapeutic schedule once RAIR occurs. The *BRAF^V600E^* mutation exhibits a high specificity for PTC, especially the classic variant, whereas it was never detected in follicular and medullary thyroid carcinoma or benign thyroid neoplasms (5).

As far as the prognostic role of *BRAF^V600E^* mutation concerned, several studies have evaluated the impact of the *BRAF^V600E^* mutation on RAI therapy but reached controversial conclusions. Some studies have shown that *BRAF^V600E^* mutation promotes iodine uptake impairment and leads to RAIR of PTC (6, 7). One possible explanation for this strong impact of *BRAF^V600E^* mutation on the non-radioiodine-avid status may be related to the recent evidence that the mutation correlates with a lower expression of sodium/iodide symporter (*NIS*) (8). Other studies have reported *BRAF^V600E^* mutation to not have a close relationship with the clinical response to RAI therapy for PTC patients (9–11). Due to inconsistent results and lack of meta-analysis to systematically review the impact of *BRAF^V600E^* mutation on RAI therapy, we present the first meta-analysis to explore the association between *BRAF^V600E^* mutation and RAI therapeutic effects in PTC by summarizing the results of different studies.

## 2 Methods

### 2.1 Publication research and eligibility criteria

This systematic review and meta-analysis was conducted following the Meta-analysis of Observational Studies in Epidemiology (MOOSE) reporting guideline (12). A comprehensive search of medical literature was conducted on studies evaluating the effect of *BRAF^V600E^* mutation on RAI therapy in patients with PTC. The following terms was used for a systematic literature search in PubMed, EMBASE, and the Science Citation Index databases: ([BRAF] OR [molecular marker]) AND ([thyroid cancer] OR [thyroid carcinoma]) AND ([radioiodine refractoriness] OR [131I refractoriness] OR [iodine-131 refractoriness]). Language restrictions were not applied. The search was performed independently by 2 researchers. The abstracts of all potential articles and related articles according to titles were reviewed. In addition, manual examination of the reference list of included articles was performed.

**Figure 1:**
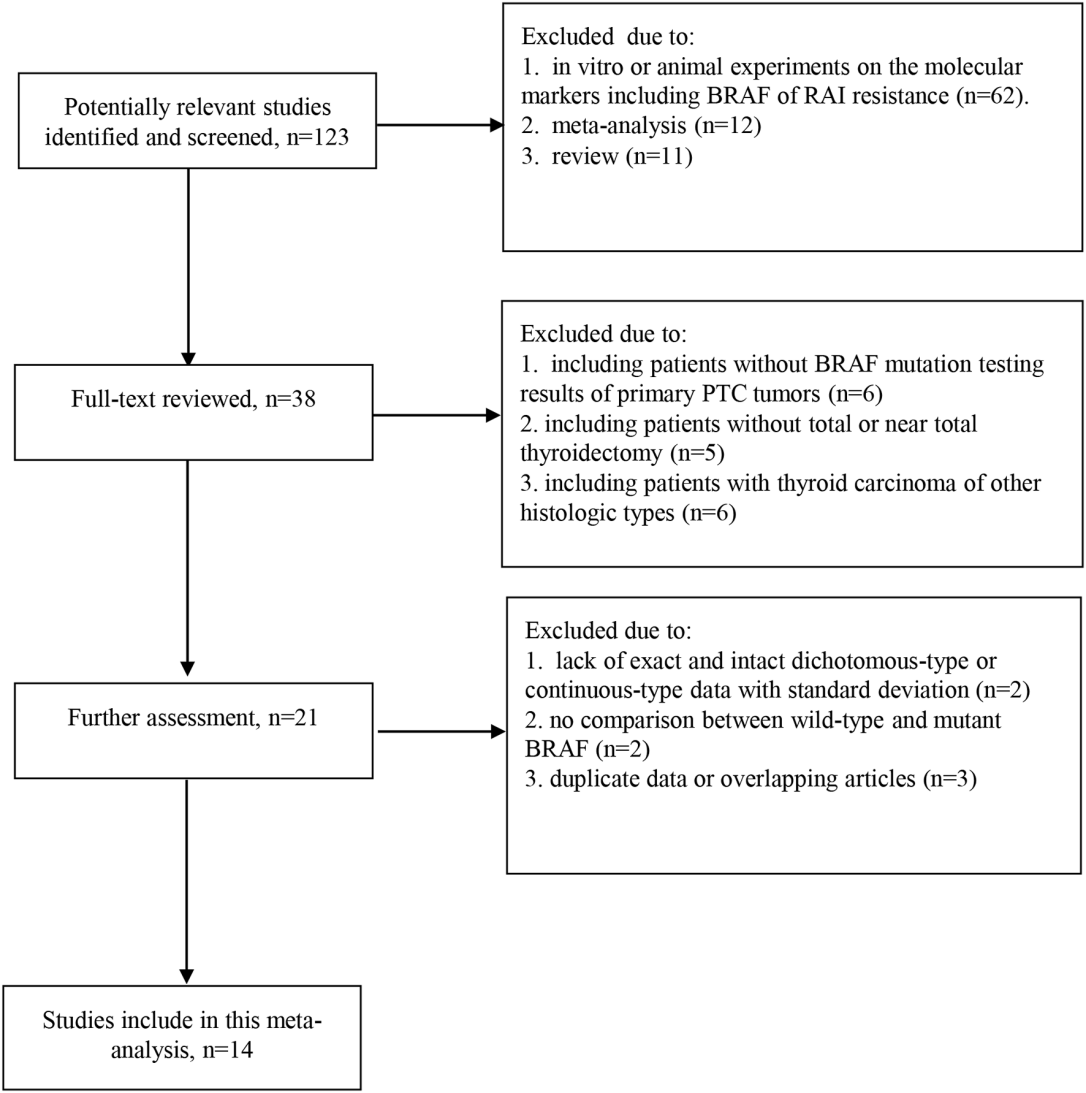
Flowchart of studies selection process for the meta-analysis.

Each article was independently assessed and included if it fulfilled the following inclusion criteria: (a) The original articles published after January 2005, (b) All of *BRAF^V600E^* mutation examined on tissues of primary tumor, (c) Comparative studies of RAI therapeutic avidity and response between wild type and mutant *BRAF* in PTC, (d) All of the PTC cases receiving “total or near total thyroidectomy + RAI therapy + L-T4 suppression therapy” which was considered as standard treatment. Studies were excluded if (a) contained no exact and intact dichotomous-type or continuous-type data with standard deviation on the therapeutic effect of RAI, or no comparison between wild-type and mutant *BRAF*; (b) *in vitro* or animal experiments on the molecular markers including *BRAF* of RAI resistance; (c) included other histologic types of thyroid carcinoma such as follicular carcinoma, medullary carcinoma, and anaplastic carcinoma. For repeated publications, the most up-to-date or informative report was included. The selection process was independently performed by 2 authors. Disagreement was resolved by consensus or by a third author. Our procedure for study screening and selection was shown in **Figure 1**.

### 2.2 Data extraction

Two investigators independently extracted and collected data using a standardized data-extraction protocol. Two authors (Bin Wang and Xiao-Xia Cen) independently assessed the quality of the included studies according to the Newcastle–Ottawa Scale (NOS) (13). The NOS includes three broad perspectives: selection (four items), comparability (two items), and exposure/outcome (three items). The maximum score for case-control and cohort studies was 9 points. Disagreements regarding methodological assessment were discussed and resolved through consensus. For each study, the following data were extracted: first author’s last name, publication year, country, enrolled population, study design, the duration of follow-up, sample size and prevalence of *BRAF* mutation.

The following data were also collected: patient characteristics (gender and age), clinicopathologic characteristics (tumor size, extrathyroidal extension, multifocality, lymph node metastases and AJCC TNM stage) and RAI therapeutic effect (^131^I uptake status, clinical response and recurrence). In the cases of incomplete required information, authors were contacted for additional information which was added as best as possible. For the assessment of ^131^I uptakes status in identified RAIR, two types were defined as follows: (1) radioiodine avid, any focal or diffuse uptake in distant metastatic lesions after excluding both contamination and physiological uptake, and (2) non radioiodine avid, negative diagnostic whole body scan results without remnant thyroid. Response to initial RAI therapy was assessed by the ATA and classified as follows(3): (1) excellent response (ER), no clinical, biochemical, or structural evidence of disease; (2) indeterminate response, nonspecific biochemical or structural findings that cannot be definitely classified as either benign or malignant; (3) biochemical incomplete response, abnormal thyroglobulin (Tg) or rising anti-Tg antibody levels in the absence of localizable disease; and (4) structurally incomplete response: persistent or newly identified loco-regional or distant metastases.

### 2.3 Statistical analysis

Data extracted from the included studies were integrated with Review Manager Software version 5.4 (Cochrane Collaborative, Oxford, UK). The results were presented as odds ratio (OR). Heterogeneity among studies was quantitatively assessed using the Q test and I^2^ statistic (14). A significant heterogeneity was defined as I^2^ > 50% or χ2-test reporting a P value < 0.05. The application of random-effects model or fixed-effects model was dependent on statistically significant heterogeneity. Sensitivity analysis was applied by removing individual studies from the data set and evaluating the effect of their removal on the pooled ORs. To further investigate potential sources of heterogeneity and the influence of study-level covariates, a random-effects meta-regression was conducted using the stata 17.0 with the metareg package. Possible publication bias was tested by Begg’s funnel plot as well as Egger’s linear regression test for the outcomes with ≥ 5 studies.

## 3 Results

### 3.1 Study selection

A total of 1360 abstracts and titles were obtained through electronic searches. Of these, 1237 abstracts were screened by the titles and abstracts and excluded. The remaining 123 records were considered relevant and full texts were reviewed in detail. Sixty-two records were excluded because the studies were *in vitro* or animal reports investigating the molecular markers including *BRAF* of RAI resistance. The remaining 61 full-text papers were deemed relevant and were examined in detail and 38 of these full-text articles were excluded (**Figure 1**). Finally, the literature search identified 14 studies (15–28) published between March 2006 and May 2023 for this meta-analysis.

### 3.2 Baseline characteristics of the included studies

In the 14 studies included in this meta-analysis, a total of 2890 PTC patients included 2603 primary PTCs and 287 recurrent PTCs. The sample size of each study ranged from 38 to 1220. The mean or median age ranged from 37 to 52 years, and the proportion of females ranged from 56.1% to 70.7%. The prevalence of *BRAF^V600E^* mutation ranged from 30.2% to 81.7%. Total thyroidectomy or near total thyroidectomy and RAI therapy were performed as initial treatment on the patients in all of the included studies. Among the included 14 studies, 13 were retrospective case-control studies and 1 was prospective cohort studies. The quality scores of the nine studies ranged from 6 to 8 with a mean of 7.2 (**Table 1**).

**Table 1:** Characteristics of the articles included.

Reference	Country	Enrolled population	Study design	Duration of follow-up	Sample size	Prevalence of BRAF mutation (%)	Quality score
Anekpuritanang 2021 (15)	Thailand	Primary PTCs	Retrospective case-control study		60	80.0%	8
Barollo 2010 (16)	Italy	Recurrent PTCs	Retrospective case-control study	9 years	50	66.0%	8
Cao 2022 (17)	China	Primary PTCs	Retrospective case-control study		126	81.7%	7
Collina 2019 (18)	Italy	Primary PTCs	Retrospective case-control study	7 years	110	43.6%	7
Elisei 2008 (19)	Italy	Primary PTCs	Retrospective case-control study	15 years	102	37.3%	6
Huang 2022 (20)	China	Primary PTCs	Retrospective case-control study		94	35.1%	8
Laschinsky 2023 (21)	Germany	Primary PTCs	Retrospective case-control study	1 years	38	63.2%	7
Li 2016 (22)	China	Primary PTCs	Retrospective case-control study	2.34 years	228	67.1%	7
Liu 2020 (23)	China	Recurrent PTCs	Retrospective case-control study		164	62.2%	7
Riesco-Eizaguirre 2006 (24)	Spain	Primary PTCs	Retrospective case-control study	3 years	67	41.8%	7
Shen 2018 (25)	China	Primary PTCs	Retrospective case-control study	3 years	512	66.0%	6
Yang 2014 (26)	China	Recurrent PTCs	Prospective cohort study		73	30.2%	8
Zhu 2019 (27)	China	Primary PTCs	Retrospective case-control study		1220	80.2%	7
Zoghlami 2014 (28)	France	Primary PTCs	Retrospective case-control study	10 years	46	43.5%	8

### 3.3 *BRAF^V600E^* mutation and demographic and clinicopathologic characteristics of PTC

**Figure 2:**
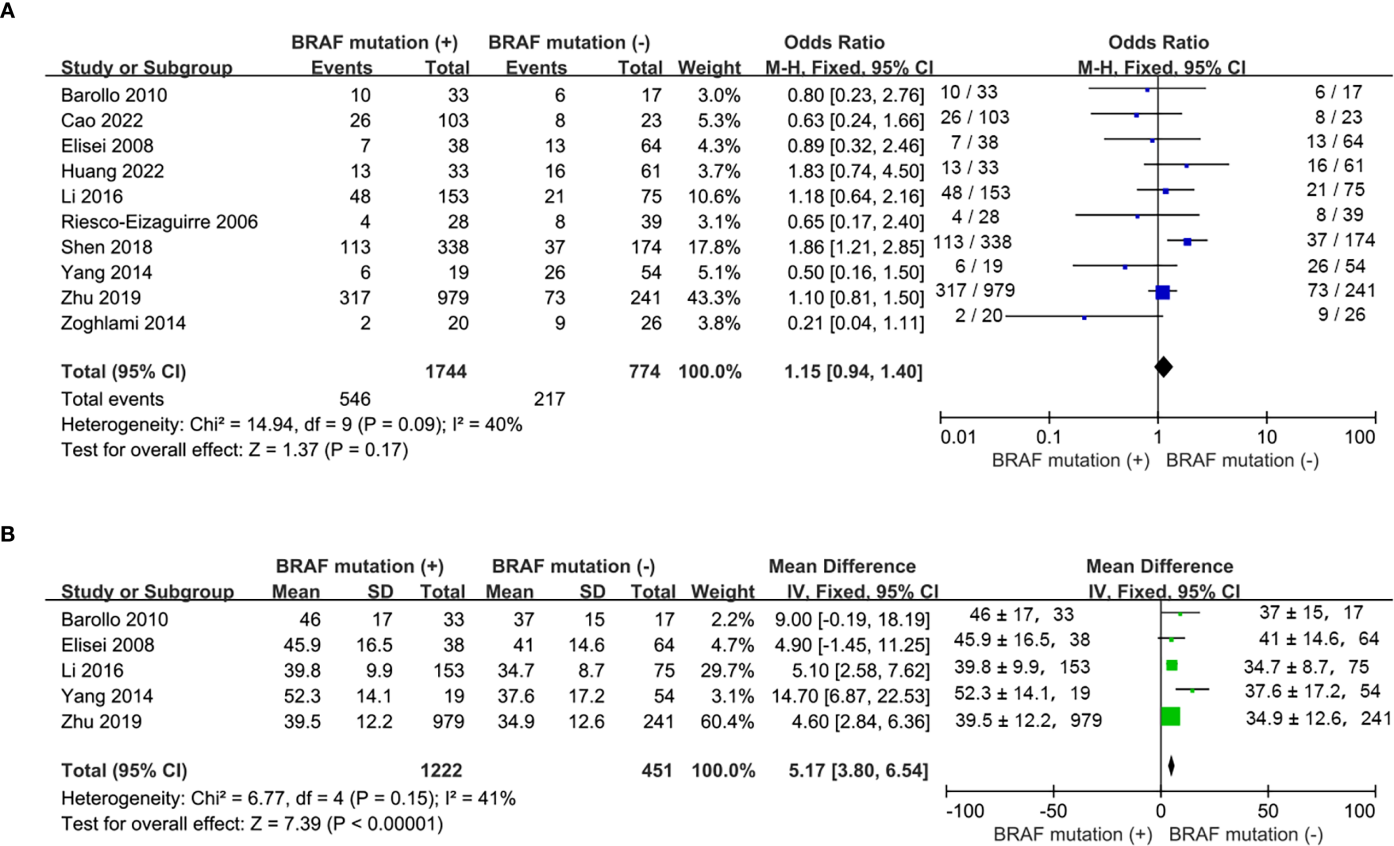
Meta-analysis of the association between demographic characteristics and BRAF^V600E^ mutation. **(A)** gender, **(B)** age.

In total, 10 studies were comparable in terms of gender (16, 17, 19, 20, 22, 24–28) and 5 studies reported mean age (16, 19, 22, 26, 27). The proportion of females was similar between mutant and wild type *BRAF* patients (OR = 1.15; P = 0.17; **Figure 2A**). Compared with the wild-type *BRAF* patients, the mean age was significantly older in the mutant *BRAF* patients (MD =5.17; P<0.00001; **Figure 2B**) There was no statistically significant heterogeneity in both analyses (gender: P for heterogeneity =0.09, I² = 40%, **Figure 2A**; age: P for heterogeneity =0.15, I² = 41%, **Figure 2B**).

**Figure 3:**
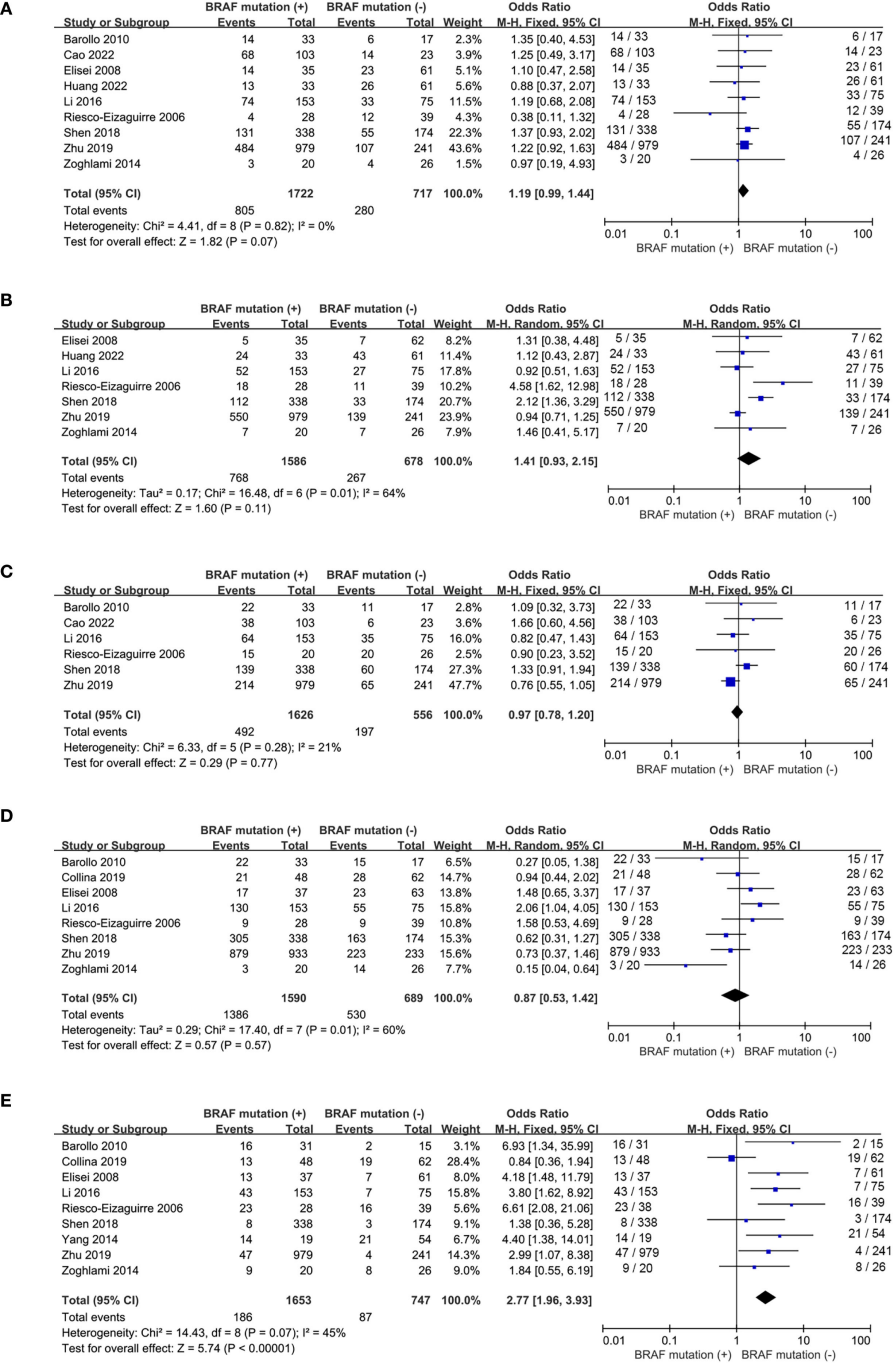
Forest plots for meta-analysis of clinicopathological characteristics associated with BRAF^V600E^ mutation. **(A)** multifocality, **(B)** extrathyroidal extension, **(C)** tumor size > 2cm, **(D)** lymph node metastases, **(E)** advanced stages.

Nine studies included (16, 17, 19, 20, 22, 24, 25, 27, 28) reported the prevalence of patients with multifocality and 7 studies included (19, 20, 22, 24, 25, 27, 28) addressed the frequency of extrathyroidal extension. No statistically significance was observed for tumor multifocality and extrathyroidal extension between wild-type and mutant *BRAF* patients (multifocality: OR, 1.19; P = 0.07; **Figure 3A**; extrathyroidal extension: OR, 1.41; P = 0.11; **Figure 3B**). No statistically significant heterogeneity existed in this meta-analysis of multifocality (P for heterogeneity =0.82, I²=0%, **Figure 3A**) whereas statistically significant heterogeneity was present in the analysis of extrathyroidal extension (P for heterogeneity =0.01, I²=64%, **Figure 3B**).

The prevalence of tumor size>2cm was analyzed in 6 studies (16, 17, 22, 24, 25, 27) and lymph node metastases was reported in 8 studies (16, 18, 19, 22, 24, 25, 27, 28). The percentages of tumor size > 2cm and lymph node metastases both failed to show significant difference between wild-type and mutant *BRAF* patients (tumor size > 2cm: OR, 0.97; P = 0.77; **Figure 3C**; lymph node metastases: OR, 0.87; P = 0.57; **Figure 3D**). However, in the 9 studies (16, 18, 19, 22, 24–28) reporting the prevalence of clinical stages III/IV according to the 8th AJCC TNM stage, the overall prevalence of advanced stages was higher in the *BRAF^V600E^* mutation patients compared to the wild-type *BRAF* patients (OR = 2.77; P<0.00001; **Figure 3E**). Statistically significant heterogeneity failed to be detected in the analysis of tumor size and advanced stages (tumor size: P for heterogeneity=0.28, I² = 21%; **Figure 3C**; stage III/IV: P for heterogeneity=0.07, I² = 45%; **Figure 3E**) whereas a high degree of study heterogeneity was observed in the analysis of lymph node metastases (P for heterogeneity =0.01, I²=60%, **Figure 3D**).

### 3.4 *BRAF* ^V600E^ mutation and curative effect of RAI therapy

The studies included in this meta-analysis employed heterogeneous methods to evaluate the therapeutic efficacy of RAI, including radioiodine avid, the response to RAI therapy, and recurrence. The definitions and assessment criteria for these endpoints, as applied in each individual study, were summarized in the **Supplementary Table S1**.

**Figure 4:**
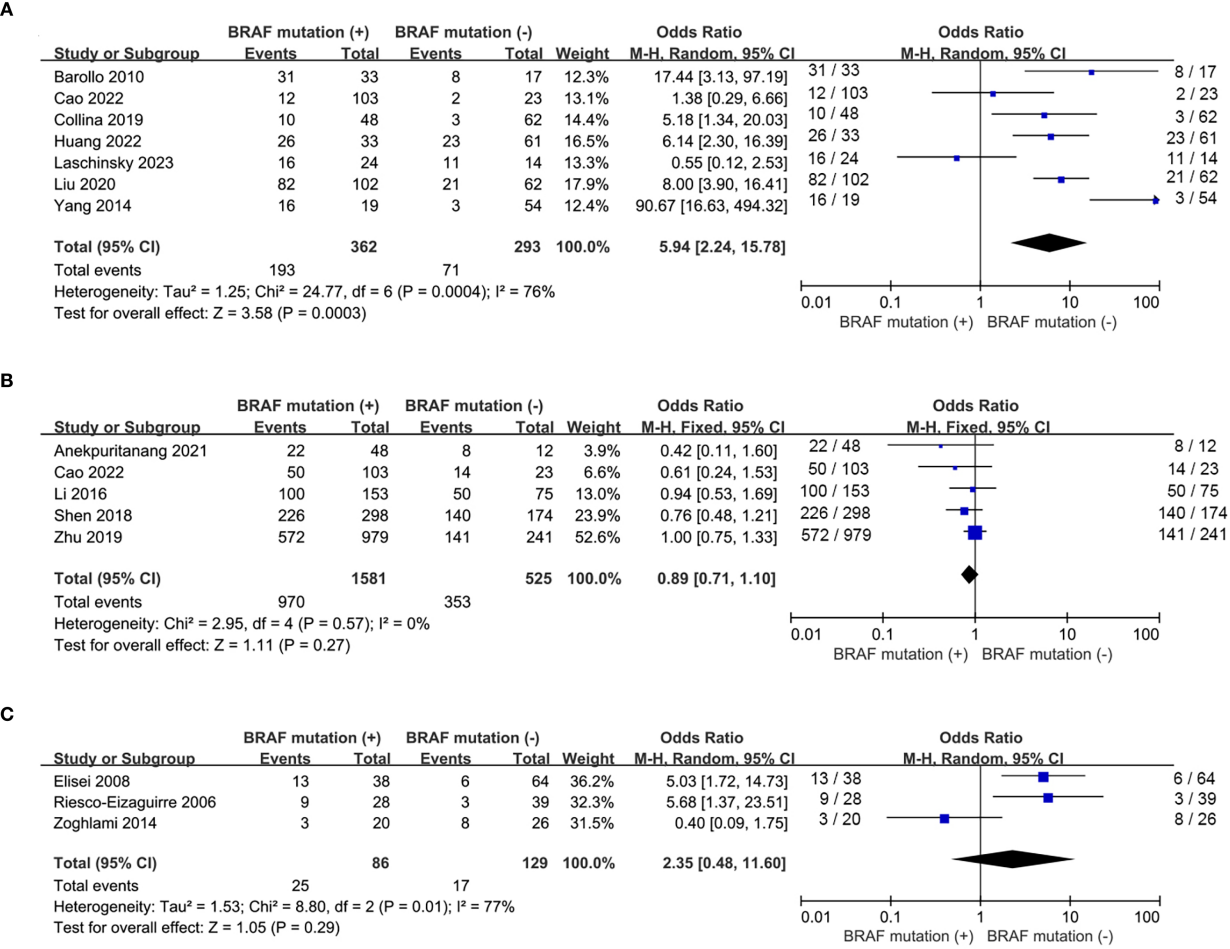
Pooled analysis of curative effect of RAI therapy according to BRAF^V600E^ mutation. **(A)** loss of iodine avidity, **(B)** ER response to RAI therapy, **(C)** recurrence after initial treatment.

**Figure 5:**
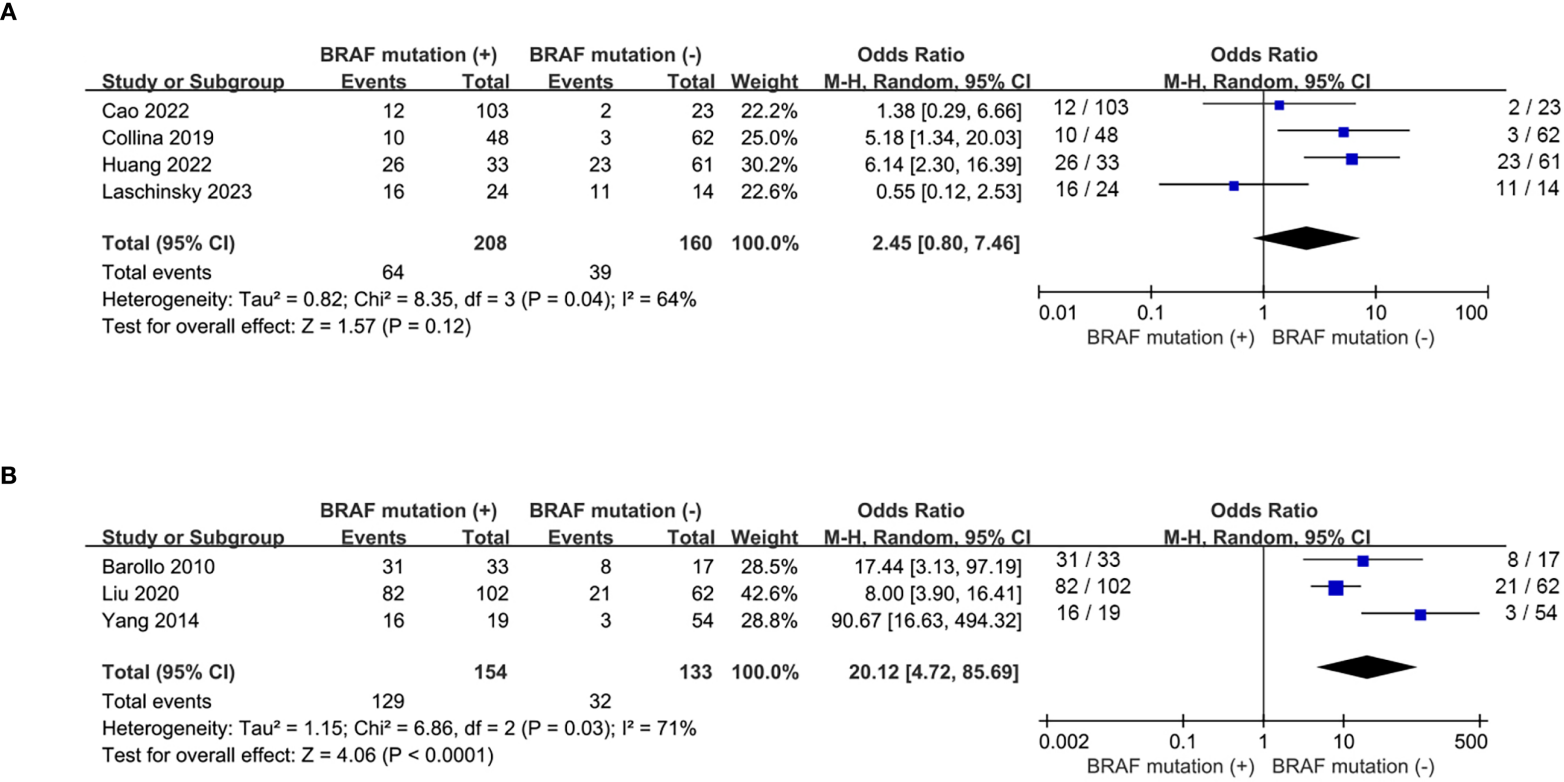
Subgroup analysis of the impact of BRAF^V600E^ mutation on loss of iodine avidity. **(A)** primary PTCs, **(B)** recurrent PTCs.

A total of 6 eligible studies (16–18, 20, 21, 23, 26) analyzed the relationship between ^131^I uptake status and *BRAF^V600E^* mutation. *BRAF^V600E^* mutation was statistically significantly associated with the loss of iodine avidity (OR = 5.94, P = 0.0003, **Figure 4A**). Statistical heterogeneity was observed among these studies (I^2^ = 76%, P = 0.0004). Given that 4 of the 7 eligible studies (17, 18, 20, 21) enrolled primary PTC patients and other 3 studies (16, 23, 26) enrolled recurrent PTC patients, a subgroup analysis should be conducted to further explore the impact of *BRAF^V600E^* mutation on RAIR in patients either primary or recurrent PTCs. The subgroup analysis of primary PTCs showed a relatively higher cumulative prevalence of RAIR in mutant *BRAF* patients compared to wild-type *BRAF* patients, but this did not reach statistical significance (OR = 2.45, P = 0.12, **Figure 5A**). In the subgroup analysis of recurrent PTCs, *BRAF^V600E^* mutation was strongly associated with RAIR (OR = 20.12, P<0.0001). Statistically significant heterogeneity still exited in both subgroup analyses (primary PTC: P for heterogeneity =0.04, I² = 64%, **Figure 5A**; recurrent PTC: P for heterogeneity =0.03, I² = 71%, **Figure 5B**).

Five studies (15, 17, 22, 25, 27) addressed the impact of *BRAF^V600E^* mutation on the response to RAI therapy. The prevalence of ER response to RAI therapy was similar between wild-type and mutant *BRAF* patients (OR = 0.89, P = 0.27, **Figure 4B**) and no significant statistical heterogeneity was detected among these studies (P for heterogeneity =0.57, I^2^ = 0%, **Figure 4B**). Recurrence after initial treatment (surgery with ^131^I ablation) was reported in 3 eligible studies (19, 24, 28). The difference was not significant between wild-type and mutant *BRAF* patients (OR = 2.35, P = 0.29, **Figure 4C**). Heterogeneity was confirmed to be high in the recurrence analysis (P for heterogeneity =0.01, I^2^ = 77%, **Figure 4C**).

### 3.5 Sensitive analysis and meta regression analysis

**Figure 6:**
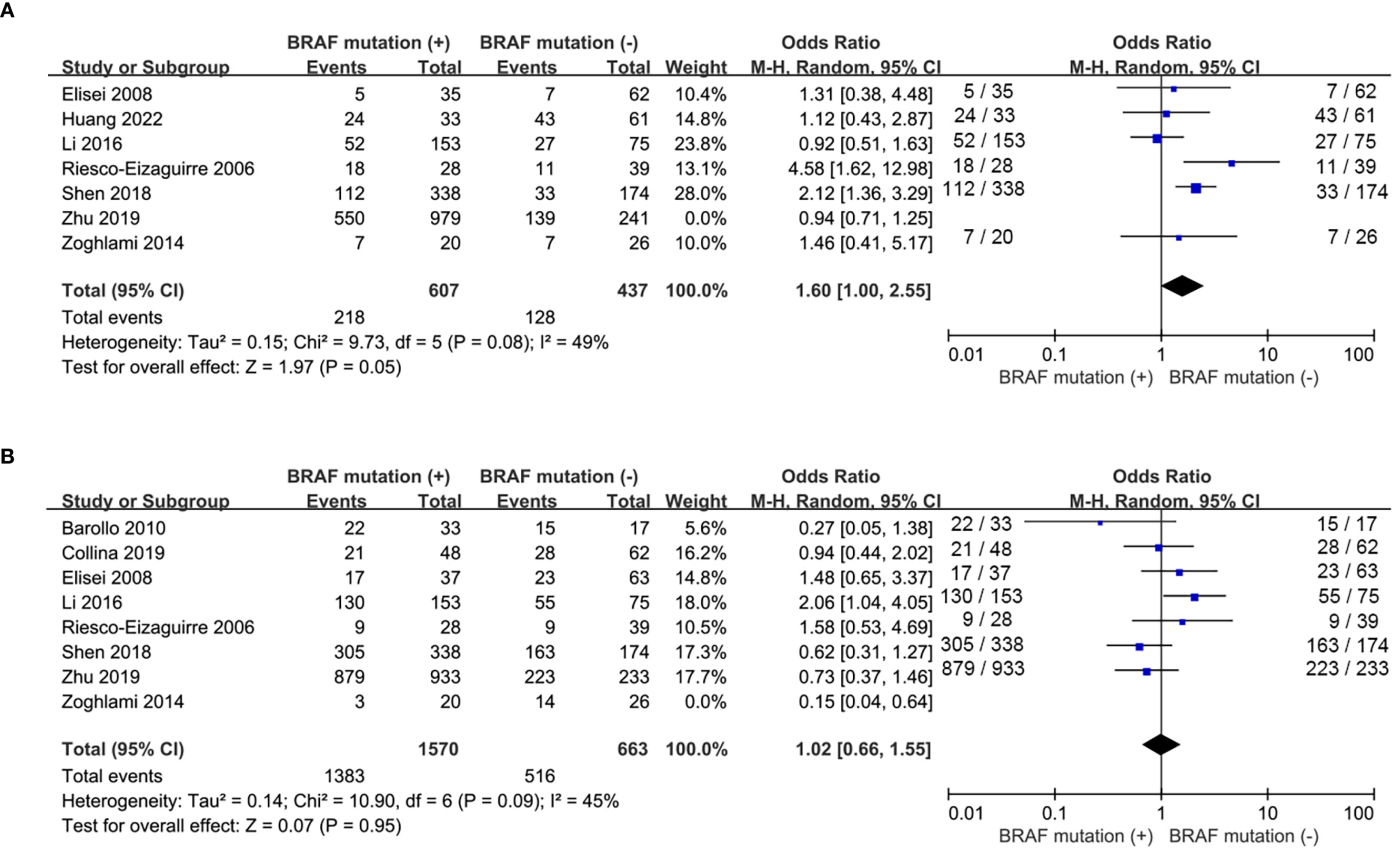
Sensitive analyses. **(A)** extrathyroidal extension **(B)** lymph node metastases.

**Figure 7:**
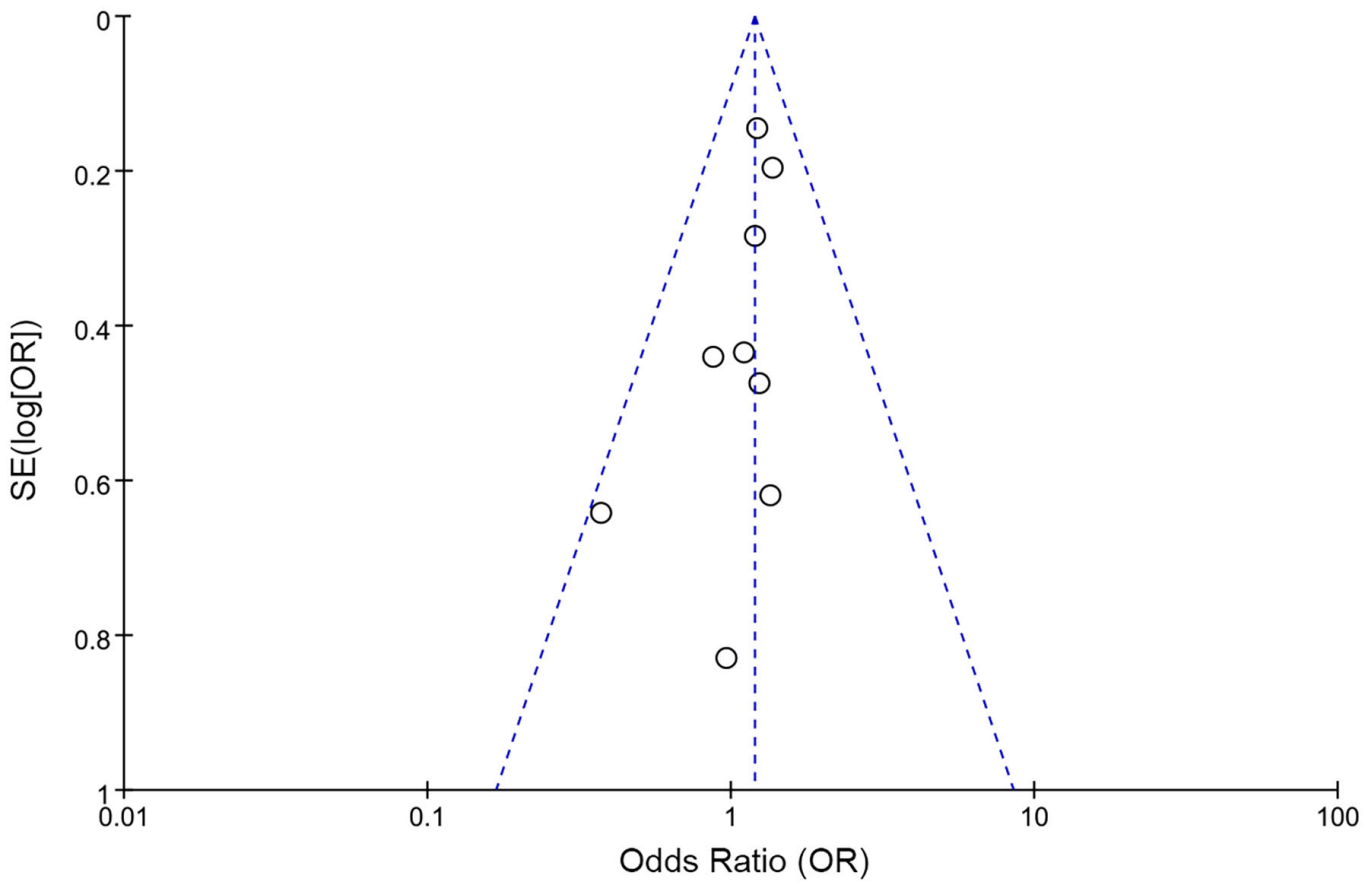
Funnel plot with Egger’s regression test for publication bias.

Analyses of extrathyroidal extension and lymph node metastasis both showed high heterogeneity (I² = 64% and 60%, respectively). As shown in **Figure 6**, sensitive analysis for extrathyroidal extension indicated that the results remained statistically non-significant (P = 0.05) after excluding the study by Zhu et al. (27), with reduced heterogeneity (I² = 49%, p = 0.08), likely due to their markedly high rate of classic PTC (95.7%) distinct from other studies. Sensitivity analysis for lymph node metastasis revealed that the results remained unchanged (P = 0.95) after removing the study by Zoghlami et al. (28), with heterogeneity resolved (I²= 45%, p = 0.09), possibly due to its unique inclusion criterion of tumor size≥10 mm. The heterogeneity of ^131^I uptake status was high (I^2^ = 78%). We performed a leave-one-out sensitivity analysis to evaluate the influence of individual studies on the pooled ORs for the relationship between ^131^I uptake status and *BRAF* mutation. The results showed that the pooled ORs and the level of heterogeneity did not change substantially after removing any single study, suggesting that the high heterogeneity was not particularly influenced by any one particular study and limited the interpretability of pooled estimates. Meta-regression indicated that geographic region (Country), sample size, and prevalence of *BRAF* mutation had no significant impact on heterogeneity for RAIR (**Table 2**). In the funnel plots and the Egger’s regression tests, there was no evidence of publication bias (**Figure 7**). Publication bias tests were not performed for recurrence (n=3) as well as both subgroup analyses (primary PTC: n=4; recurrent PTC: n=4) due to the small number of studies included.

**Table 2:** Meta regression of heterogeneity sources in radioiodine avid analysis.

Logrr	Coefficient	Std. err.	T	P>| t |	[95% conf. interval]
Region (country)	-0.661	1.772	-0.37	0.734	-6.303 - 4.981
Sample size	-0.001	0.021	-0.03	0.979	-0.068 - 0.067
prevalence of *BRAF* mutation	-4.812	4.198	-1.15	0.335	-18.173 - 8.548
_cons	5.397	4.020	1.34	0.272	-7.395 - 18.189

## 4 Discussion

RAI is an important treatment for PTC after thyroidectomy, including remnant ablation, adjuvant therapy, and therapy for persistent/recurrent disease. RAI adjuvant therapy is recommended for high-risk level PTC patients and should be considered in intermediate-risk level PTC patients after total thyroidectomy according to ATA risk of recurrence risk stratification (3). However, there remains a proportion of RAIR-PTC patients. Despite RAI therapy, these patients face disease progression and relapse.

The *BRAF* status, as a relatively sensitive prognostic marker for PTC, has a potential value in isolation as an aid to risk stratification based on clinicopathological classification. However, its independent prognostic value remains uncertain and not yet definitive for clinical decision-making. Some studies confirmed that *BRAF* was related with poor prognosis and high risk features (5, 6). Conversely, Lee’s et al. study showed negative *BRAF^V600E^* tumor was associated with more aggressive behavior with a high risk of developing distant metastasis in patients with PTC (29). RAIR-PTCs refers to PTCs that indicated a poor or no objective response to RAI treatment. Three distinct mechanisms underlie this refractoriness: RAI insufficiency, resistance and indifference. RAI insufficiency results from an inadequate radiation-absorbed dose delivered to the tumor. RAI-resistance involves a biologic imbalance between radiation-induced cytolethal damage and cellular repair capacity. RAI indifference, the most prevalent and critical mechanism, is characterized by absent avidity or responsiveness to RAI due to impaired functional differentiation, primarily driven by constitutive MAPK–ERK pathway activation, usually via *BRAF* mutation (30). V600E is a mutation of the *BRAF* gene in which valine (V) is substituted by glutamic acid (E) at amino acid 600. AlphaFold-predicted structure of *BRAF*, with the location of V600E mutation in the dark blue region was shown in **Supplementary Figure S1**. *T*he *BRAF* gene is a key component of the MAPK signal pathway, which regulates cell growth, differentiation and survival, and mutation in the *BRAF* gene can lead to sustained activation of this pathway, resulting in uncontrolled cell proliferation and tumor development (31). The mutation in the *BRAF* gene plays an important role in the mechanisms underlying RAIR in PTC. Preclinical studies have demonstrated that the presence of *BRAF^V600E^* mutation significantly reduces *NIS* expression and RAI uptake (32). *BRAF^V600E^* mutation has been confirmed to downregalute the transcription of the *NIS* gene (33), which reduces the binding of paired box 8 to the upstream enhancer of the *NIS* through TGFβ-SMAD3 and MEK-ERK signaling pathways, leading to a reduction in the level of *NIS* expression and its localization at the cell membrane surface (34, 35). It has also been reported that *BRAF^V600E^* mutation can decrease RAI uptake by promoting the histones deacetylation of *NIS* promoter through *HDAC (* 36). Clinical data has demonstrated that the mRNA levels of *NIS* in the *BRAF* mutation-negative group were five times higher than those in the *BRAF* mutation positive group (37). Mutations in *SWI/SNF* genes may be another underlying mechanism through which *BRAF^V600E^* mutation leads to reduced responsiveness to RAI treatment. Loss of individual *SWI/SNF* subunits in *BRAF^V600E^* mutation tumors leads to a repressive chromatin state that cannot be reversed by MAPK pathway blockade, rendering them insensitive to its redifferentiation effects (38).

Although *BRAF^V600E^* mutation is mechanistically linked to impaired ^131^I uptake, its association with the curative efficacy of RAI therapy remains controversial in clinical studies. The correlation of *BRAF^V600E^* mutation with RAIR of PTC is controversial in clinical studies. It has been shown that in patients with PTC there is a higher incidence of distant metastases without ^131^I uptake in the *BRAF^V600E^* mutation positive group than in the negative group (84.2% vs. 5.6%) (39). Another research demonstrated that the sensitivity and specificity of predicting ^131^I non-uptake in distant metastatic foci of PTC using the *BRAF^V600E^* mutation were 84.2% and 94.4%, respectively (26). The study of Ge et al. reported that *BRAF^V600E^* mutation is closely associated to post-RAI serum Tg elevation in PTCs (40). However, other studies have also reported inconsistent results. The status of *BRAF^V600E^* did not affect the clinical response of RAI therapy in patients with papillary thyroid microcarcinoma at intermediate to high recurrence risk and in low-intermediate risk recurrent PTC (10, 11). Among the patients with tumors measuring 10 mm or larger in diameter, the recurrence rate after RAI therapy had no significant difference between wild-type and mutant *BRAF* patients during the follow-up of 10 years (28). In this meta-analysis, some poor prognostic factors such as age, extrathyroidal extension and tumor size, cannot be ignored as confounding factors in the evaluation of the curative effect of RAI therapy. However, this meta-analysis of aggregate data from the including studies did not permit multivariate adjustment for key prognostic variables due to the unavailability of original individual-patient data. Therefore, the evaluation of *BRAF ^V600E^* as an independent predictor of RAIR should be approached with caution.

A total of 14 studies with 2890 PTC patients were included in this meta-analysis. For the first time, the effect of *BRAF^V600E^* mutation on RAI therapy for PTC was pooled analyzed. Among the included studies, the efficacy of RAI therapy was assessed using three primary endpoints: RAI avidity, treatment response, and recurrence. The lack of standardized definitions for these endpoints led to considerable variation in the reported outcomes. Results showed that *BRAF^V600E^* mutation was statistically significantly associated with ^131^I avidity loss (OR = 5.94, P = 0.0003), which was in line with the findings reported in another systematic review of Luo et al. (41). Unlike the study of Luo et al. (41), which combined follicular and Hürthle cell thyroid carcinomas, the purpose of our PTC-specific analysis is to provide direct insights into the management of *BRAF*-mutant PTC. It was worth mentioning that *BRAF^V600E^* mutation was associated with RAI resistance only in recurrent PTCs, while there was no statistical significance in primary PTCs in subgroup analyses in this meta-analysis. Metastases often exhibit more aggressive histopathological features compared to the primary tumor. Examples include histological progression from tall cell variant of PTC (TCV) to anaplastic thyroid carcinoma (ATC), from HCC to poorly differentiated carcinoma (PDC), or from PTC to TCV and PDC (42). Great histological plasticity between primary and recurrent metastatic lesions of PTCs may account for the different roles of *BRAF^V600E^* mutation in RAI resistance (42). The incidence of ER response to RAI treatment was comparable (OR = 0.89, P = 0.27) and there was no statistically significant difference in recurrence rates following initial treatment (OR = 2.39, P = 0.29) between wild-type and mutant *BRAF* patients in our study. The observed discrepancies in the role of *BRAF^V600E^* mutation may be attributed to incomplete overlap among the three patient cohorts: those with RAIR, non-ER, and recurrent disease. RAIR, which also described as loss of iodine avidity in the results, is evaluated through radioiodine concentration and observing metastatic disease progresses (3, 43). The criteria for determining tumor recurrence and treatment response, in addition to post-RAI scintigraphy results, also combines with serum thyroglobulin (Tg) levels and cytological evidence (3, 28). There may be a population of positive *BRAF^V600E^* mutation who are RAIR but show no other objective evidence of non-ER or recurrence. This distinct patient distribution pattern directly results to relatively fewer patients with *BRAF^V600E^* mutation in the non-ER and recurrence groups compared with the ^131^I avidity loss group, leading to the negative results of *BRAF^V600E^* mutation in analyses. And because of the difference in follow-up duration, it cannot be sure whether patients in the ER group and the RAI avidity group will have a recurrence or loss of radioiodine avidity in the future.

The heterogeneity is high in ^131^I uptake status anylasis and recurrence analysis(I^2^ = 76%; I^2^ = 77%). The potential sources of this heterogeneity may include variations in follow-up duration, the diagnostic criteria for ^131^I avidity, differences in PTC subtypes distribution, RAIR definition, radiation dose or methodologies used for *BRAF* mutation testing. The follow-up duration in the retrospective researches ranged from 1 year to 10.1 years. A critical point that should not be overlooked is the substantial variation in biological characteristics among pathological subtypes of PTC. These differences significantly influence the prognosis of PTC. Some studies have demonstrated that tall cell variant PTC, sclerosing diffuse PTC, hobnail variant PTC are more likely to significantly increase the risk of resistance to RAI therapy (41). Unfortunately, due to the limited number of studies and the lack of individual patient data, we could not perform meta-regression to definitively identify these sources.

This meta-analysis represents the first comprehensive summary and analysis of the impact of *BRAF^V600E^* mutation on the efficacy of RAI therapy in PTC. However, several limitations must be acknowledged. Variability in follow-up duration and histopathological classification maybe contributed to significant heterogeneity across the studies. Moreover, pooled analyses could not adjust for key confounders including age, tumor size, extrathyroidal extension, and initial lymph node metastasis status, due to insufficient primary data. Thus, while *BRAF^V600E^* correlates with RAIR, its independent prognostic value requires confirmation in individual-patient-data meta-analyses. Consequently, high-quality prospective studies remain necessary to definitively address this clinical question. Finally, the ability of funnel plots and Egger’s test to reliably detect publication bias is reduced due to the limited number of studies.

## 5 Conclusion

This meta-analysis provides evidence that the mutation of *BRAF^V600E^* is significantly associated with the occurrence of RAIR among PTC patients. However, the potential impact of *BRAF^V600E^* mutation on clinical response to RAI therapy and disease recurrence warrants further investigation through well-designed prospective studies.

## Data Availability

The original contributions presented in the study are included in the article/**Supplementary Material**. Further inquiries can be directed to the corresponding author/s.

## Supplementary material

Supplementary Table 1 Definitions and assessment criteria for key outcomes across included studies. RAIR, radioactive iodine refractoriness; RAI, Radioactive iodine; Tg, thyroglobulin; Anti-Tg, thyroglobulin antibody; PTC, papillary thyroid carcinoma; rhTSH, recombinant human thyrotropin; WBS, ^131^I whole-body scan.

Supplementary Figure 1 AlphaFold-predicted structure of BRAF^V600E^ protein.
