# Supplementary material for: The effect of BRAFV600E mutation on radioiodine therapy in patients with papillary thyroid carcinoma: a meta-analysis and systematic review
Source: Front Endocrinol (Lausanne). 2025 Sep 23;16:1665545. doi: 10.3389/fendo.2025.1665545 (PMC12500461; doi:10.3389/fendo.2025.1665545)
Supplement: Supplementary Table 1 — Definitions and assessment criteria for key outcomes across included studies. RAIR, radioactive iodine refractoriness; RAI, Radioactive iodine; Tg, thyroglobulin; Anti-Tg, thyroglobulin antibody; PTC, papillary thyroid carcinoma; rhTSH, recombinant human thyrotropin; WBS, 131I whole-body scan. [file Table1.docx]

**Table S1** Definitions and assessment criteria for key outcomes across included studies

| **Reference** | **RAIR** | **Non-excellent ER** | **Recurrence** |
| --- | --- | --- | --- |
| Anekpuritanang 2021 (15) |  | Not mentioned. |  |
| Barollo 2010 (16) | Not mentioned. |  |  |
| Cao 2022 (17) | The malignant/ metastatic tissue demonstrates absent or only partial radioiodine avidity; lost the ability to concentrate RAI after previous evidence of RAI-avid disease; progresses despite significant concentration of RAI. | Exist clinical, biochemical, or structural evidence of disease. |  |
| Collina 2019 (18) | Lack of RAI uptake on post-treatment scan (after a >30 mCi therapeutic activity) despite rhTSH stimulation, or on a diagnostic WBS with structurally confirmed disease, or by lesional dosimetry (i.e., delivered RAI dose to metastatic foci <8000 cGy); Continued disease progression, despite cumulative RAI-administered activities >500–600 mCi. |  |  |
| Elisei 2008 (19) |  |  | Neck ultrasound and diagnostic WBS were positive, as well as when serum Tg and anti-Tg were detectable in either hypothyroidism or after rhTSH stimulation. |
| Huang 2022 (20) | Not mentioned. | Not all lesions disappeared and/or elevated serum Tg level |  |
| Laschinsky 2023 (21) | Not mentioned. |  |  |
| Li 2016 (22) |  | Positive imaging and a suppressed Tg ≥ 0.2 ng/mL or a stimulated Tg ≥1.0 ng/mL. |  |
| Liu 2020 (23) | No RAI uptake in one or more lesions of recurrent PTC on whole-body RAI scans |  |  |
| Riesco-Eizaguirre 2006 (24) |  |  | Any elevation of serum Tg associated with positive WBS and/or any other abnormal imaging study |
| Shen 2018 (25) |  | Exist clinical, biochemical, or structural evidence of disease. |  |
| Yang 2014 (26) | Negative WBS results without remnant thyroid but structural chest CT or x-ray findings on elevated Tg level. |  |  |
| Zhu 2019 (27) |  | Exist clinical, biochemical, or structural evidence of disease. |  |
| Zoghlami 2014 (28) | patients with no RAI uptake or who progressed during the months following RAI therapy or who presented persistent disease after a cumulative dose of 600 mCi. |  | elevated serum Tg with suspicious ultrasound image with confirmation of the diagnosis by fine needle aspiration cytology or positive radioiodine WBS |

RAIR: radioactive iodine refractoriness; RAI: Radioactive iodine; Tg: thyroglobulin; Anti-Tg: thyroglobulin antibody; PTC: papillary thyroid carcinoma; rhTSH: recombinant human thyrotropin; WBS: ^131^I whole-body scan
