# Supplementary figures and images for: The effect of BRAFV600E mutation on radioiodine therapy in patients with papillary thyroid carcinoma: a meta-analysis and systematic review
Source: Front Endocrinol (Lausanne). 2025 Sep 23;16:1665545. doi: 10.3389/fendo.2025.1665545 (PMC12500461; doi:10.3389/fendo.2025.1665545)

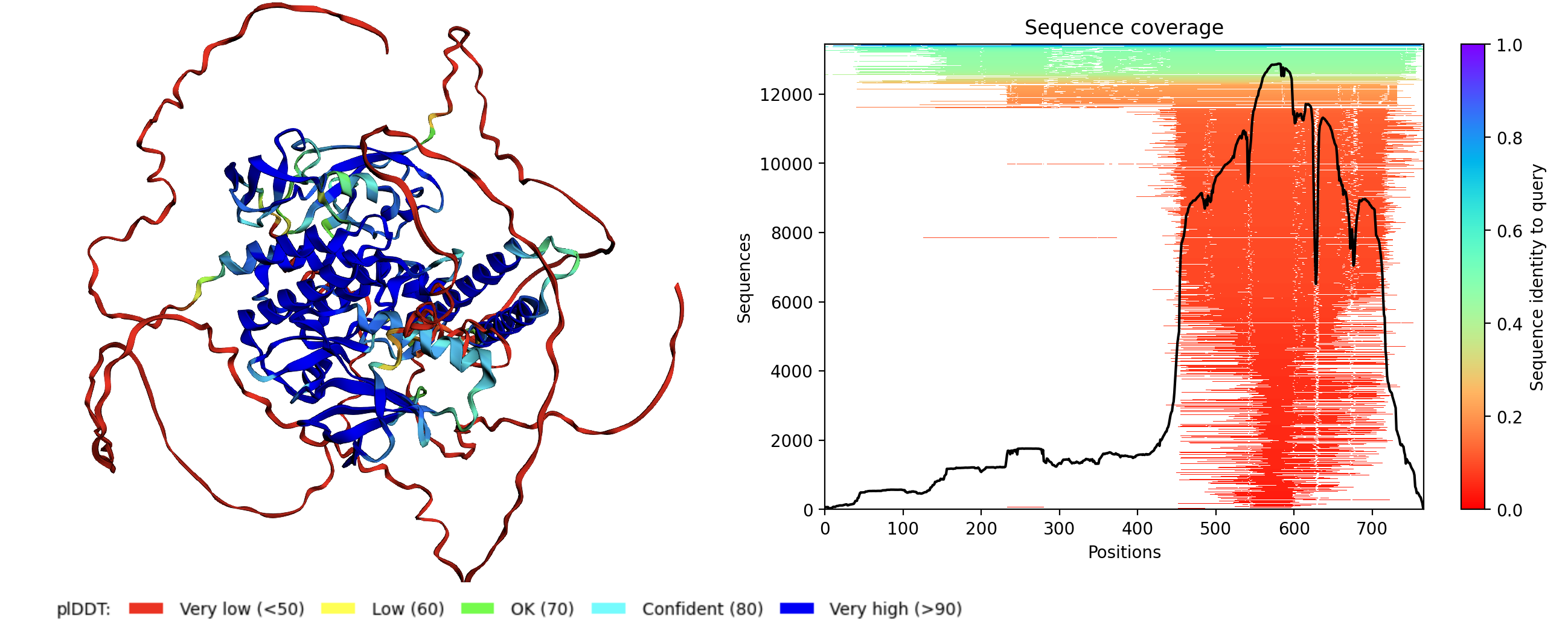

Supplement: Supplementary Figure 1 — AlphaFold-predicted structure of BRAFV600E protein. [file Image1.tif]
